# Pigment Epithelium Derived Factor Inhibits the Growth of Human Endometrial Implants in Nude Mice and of Ovarian Endometriotic Stromal Cells *In Vitro*

**DOI:** 10.1371/journal.pone.0045223

**Published:** 2012-09-18

**Authors:** Yanmei Sun, Xuan Che, Libo Zhu, Mengdan Zhao, Guofang Fu, Xiufeng Huang, Hong Xu, Fuqiang Hu, Xinmei Zhang

**Affiliations:** 1 Women’s Hospital, School of Medicine, Zhejiang University, Hangzhou, People’s Republic of China; 2 College of Pharmaceutical Science, Zhejiang University, Hangzhou, People’s Republic of China; Rutgers University, United States of America

## Abstract

Angiogenesis is a prerequisite for the formation and development of endometriosis. Pigment epithelium derived factor (PEDF) is a natural inhibitor of angiogenesis. We previously demonstrated a reduction of PEDF in the peritoneal fluid, serum and endometriotic lesions from women with endometriosis compared with women without endometriosis. Here, we aim to investigate the inhibitory effect of PEDF on human endometriotic cells *in vivo* and *in vitro*. We found that PEDF markedly inhibited the growth of human endometrial implants in nude mice and of ovarian endometriotic stromal cells *in vitro* by up-regulating PEDF expression and down-regulating vascular endothelial growth factor (VEGF) expression. Moreover, apoptotic index was significantly increased in endometriotic lesions *in vivo* and endometriotic stromal cells *in vitro* when treated with PEDF. In mice treated with PEDF, decreased microvessel density labeled by Von Willebrand factor but not by α-Smooth Muscle Actin was observed in endometriotic lesions. And it showed no increase in PEDF expression of the ovary and uterus tissues. These findings suggest that PEDF gene therapy may be a new treatment for endometriosis.

## Introduction

Endometriosis is defined by the presence of functional endometrium outside the uterine cavity that results in dysmenorrhea, dyspareunia, pelvic pain, and infertility [1]. Its mechanisms and pathophysiology are not completely understood, and there are few effective medical treatments. Many drugs used for the treatment of endometriosis, inhibit estrogen synthesis and its actions, including progesterone, danazol, gonadotropin-releasing hormone agonist (GnRH-a) [2], [3]. However, side effects limit long-term treatment and new medications with acceptable side effects will be helpful. Recently, more attention has focused on anti-angiogenic therapy since neovascularization is thought to be a prerequisite for the survival and growth of ectopic endometrium [4], [5]. Some anti-angiogenic factors, including endostatin, and selective cyclooxygenase-2 (COX-2) inhibitors, have proven to be effective in inhibiting the growth of endometriotic lesions in vitro and in vivo [6], [7].

Pigment epithelium derived factor (PEDF) is a 50-kDa secreted glycoprotein isolated from conditioned medium of cultured human fetal retinal pigment epithelial cells [8]–[12]. PEDF inhibits angiogenesis through endothelial cell apoptosis, and a variety of angiogenesis-related factors [13]–[16]. PEDF is active against vascular endothelial growth factor (VEGF), basic fibroblast growth factor (bFGF) and interleukin-8 (IL-8) [17]. PEDF also inhibits endothelial cell migration in a dose-dependent manner in vitro, and suppresses endothelial cell migration induced by other angiogenic factors such as VEGF [18]–[20]. In addition, PEDF can selectively inhibit the formation of new vessels from endothelial cells but does not appear to harm existing vascular structures [21]. Reduced PEDF expression has been demonstrated in a variety of cancers [22], [23], although there is high PEDF expression in malignant melanoma, ocular melanoma, hepatocellular carcinoma, Wilm’s tumor and prostate cancer [24]–[28]. PEDF is a potential therapeutic target in angiogenesis-dependent conditions.

**Figure 1:**
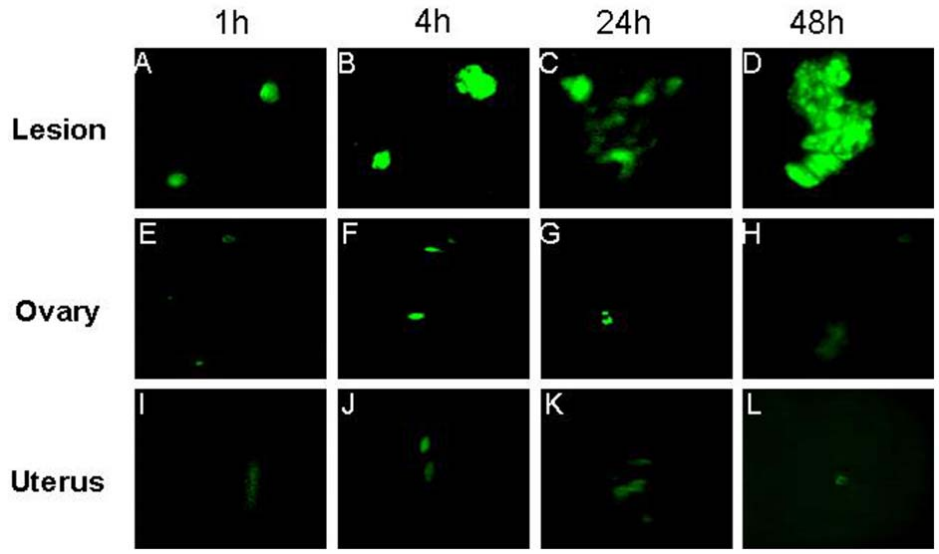
The distribution of FITC labeled CSO-SA/PEDF in endometriosis lesions (a-d), ovary (e-h) and uterus (i-l) at different time points (1, 4, 24, 48 h).

**Figure 2:**
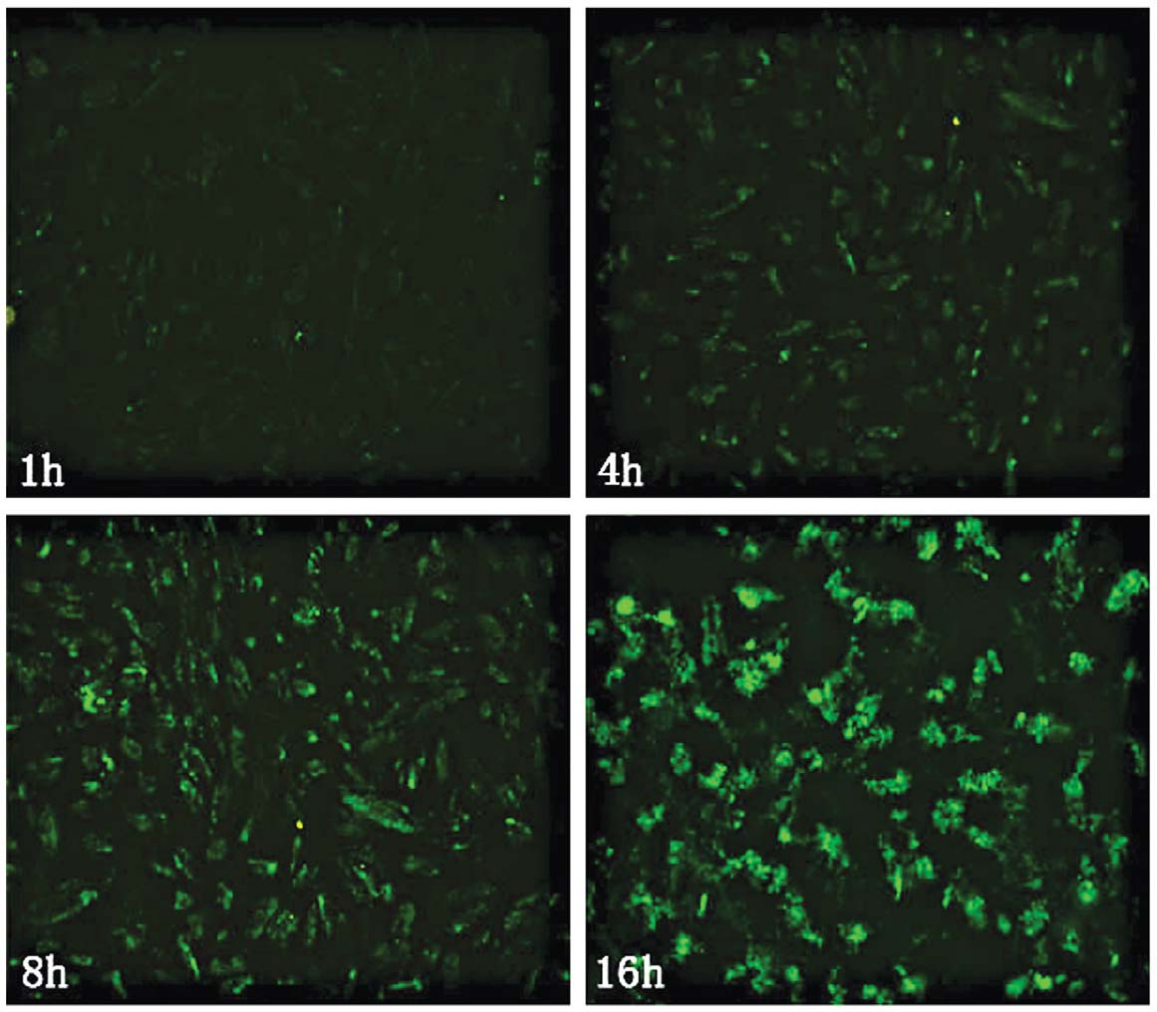
The distribution of CSO-SA/PEDF-FITC in ESCs after transfection for 1, 4, 8 and 16 h.

Recently, we found that concentrations of PEDF in serum and peritoneal fluid in women with endometriosis are decreased, and correlated with the severity and type of the disease [29], [30], and that PEDF expression is low in endometriotic lesions in women with endometriosis and in a rat model of endometriosis (unpublished results). This provides a clue that PEDF may be used as a treatment for endometriosis. In a preliminary study, we used PEDF gene to treat endometriosis induced by auto-transplantation of uterine tissue in Sprague-Dawley (SD) rats, and found that PEDF inhibited the development of endometriotic lesions [31]. However, rat endometrium is different from human endometrium in some important respects including its infiltration by immune and inflammatory cells [32]. Therefore, in the present study, we aimed to further investigate the inhibitory effect of PEDF on human endometriotic cells in vivo and in vitro.

**Figure 3:**
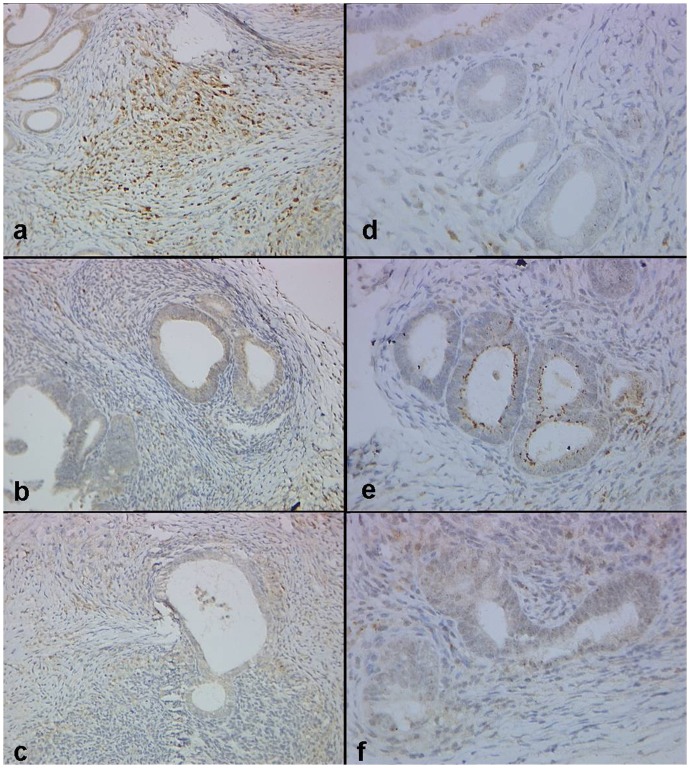
The expression of VEGF (a–c) and PEDF (d–f) in implants of control, PEDF-1 and PEDF-5 groups in nude mice. It shows that CSO-SA/PEDF treatment increases the PEDF expression both in PEDF-1 (b) and PEDF-5 (c) groups significantly compared to controls (a), while decreases the VEGF expression in PEDF-1 (e) group significantly compared to controls (d). (Original magnification ×400).

## Materials and Methods

### Surgical Induction of Endometriosis in Nude Mice

Mature, female, athymic, nude mice (nu/nu-BALB/c) aged 6–8 weeks, weighing 18–20 g were housed in micro-isolator cages in a barrier facility at a monitored ambient temperature of 22 0C. Mice were maintained in regulated, 12∶12 hour light-dark cycles, with free access to laboratory chow and water ad libitum. This study was carried out in strict accordance with the recommendations in the Guide for the Care and Use of Laboratory Animals of the National Institutes of Health. The protocol was approved by the Committee on the Ethics of Animal Experiments of Zhejiang University (Permit Number: 85–23). All surgery was performed under sodium pentobarbital anesthesia, and all efforts were made to minimize suffering.

**Table 1:** PEDF and VEGF expressions in endometriotic lesions in the treated and control groups.

Groups	VEGF		PEDF	
	stroma	glands	stroma	glands
Control	8.75±2.22	6.35±1.71	4.50±0.58	4.25±1.89
PEDF-1#	3.50±0.55*	4.03±0.93*	7.83±2.56*	9.43±3.15*
PEDF-5	6.00±1.91	6.03±1.51	7.14±2.11*	9.00±2.45*

# PEDF-1 and 5: Mice treated with PEDF gene on day 1 and 5 after transplantation.

* Indicate significant difference (p*<*0.05, versus control).

**Figure 4:**
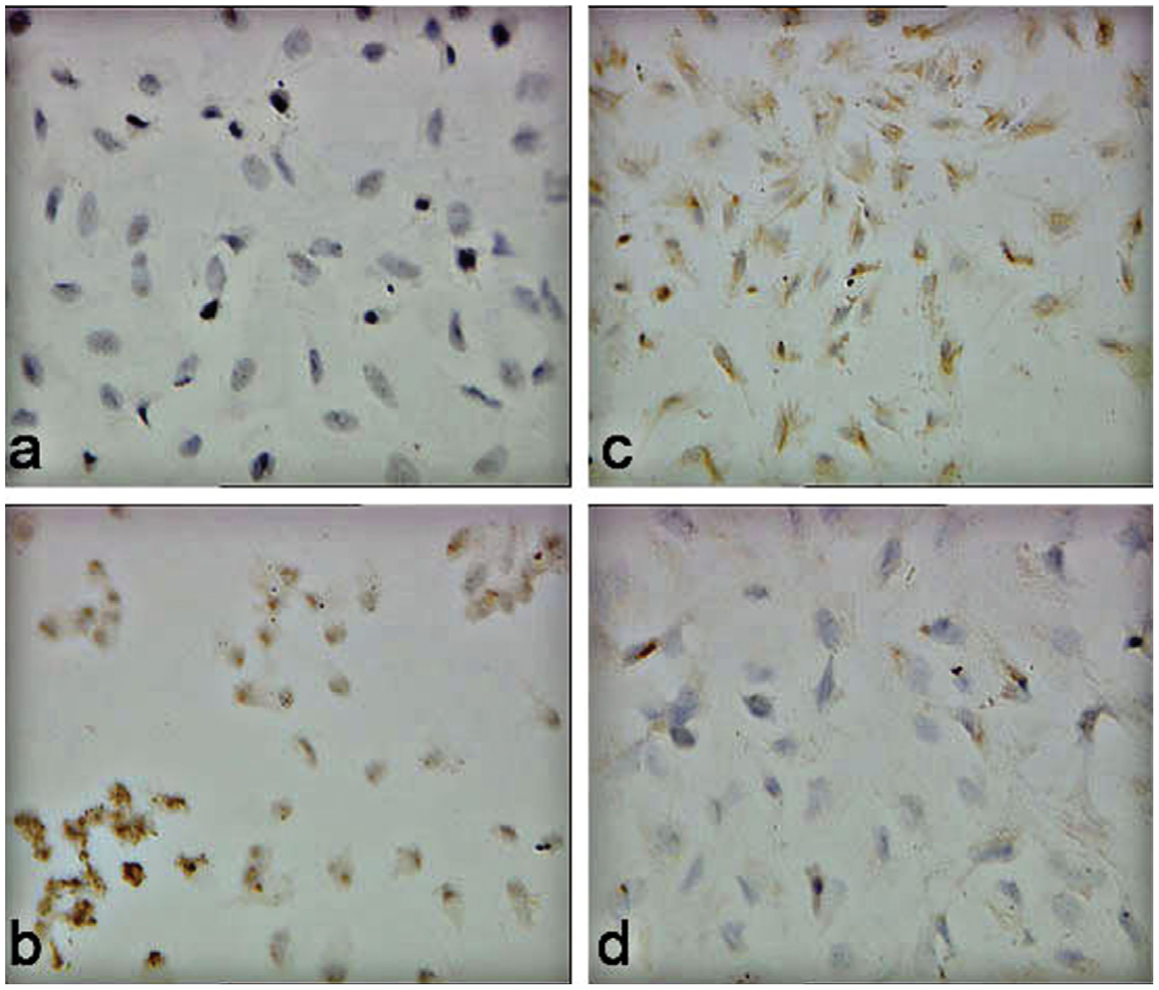
Immunocytochemistry analysis on the expression of PEDF (a, c) and VEGF (b, d) in ECSC. It shows that CSO-SA/PEDF treatment for 48 h (c) in ECSC increases the PEDF expression significantly compared to controls (a), while decreases the VEGF expression significantly compared to controls (d). (original magnification ×400).

Proliferative endometrial tissues were obtained from 7 patients with cervical intraepithelial neoplasia (CIN) III who underwent total hysterectomy (mean age 31, range 27–35 years). No patients received hormonal therapy in the 6 months before surgery. All women gave their written informed consent. The ethics committee of Women’s Hospital, School of Medicine, Zhejiang University, Hangzhou, Zhejiang, People’s Republic of China approved this study (No. 20100010).

The endometriosis model in mice was established using previously-described techniques with minor modifications [33]. Briefly, endometrial tissue in serum-free DMEM/Ham’s F-12 culture medium (Gibco BRL, Gaithersberg, MD, USA) was placed on ice and cut into 5×5 mm^2^ fragments. Each mouse was implanted subcutaneously with 3–4 endometrial fragments in the flank under general anesthesia. After transplantation, all mice were treated with 17-β-E2 (20 µg) via intramuscular injection every 5 days. One piece of the endometrial tissue was fixed in 10% formalin, dehydrated, and embedded in paraffin. Sections of 4 µm thickness were stained with hematoxylin and eosin (HE) for histologic examination.

**Figure 5:**
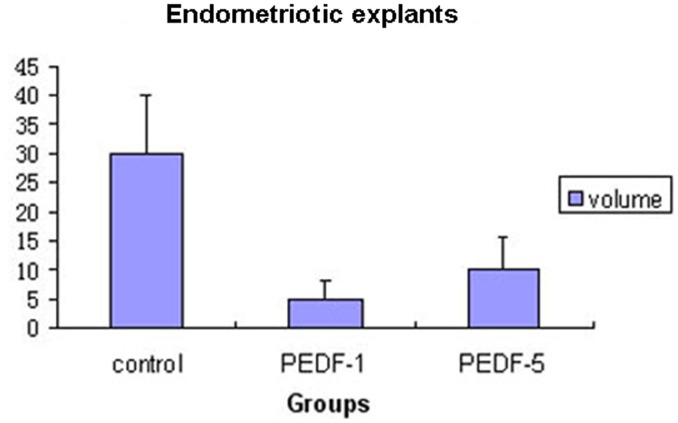
The mean of volumes of the endometriosis foci in each group (PEDF-1: CSO-SA/PEDF group when treatment was initiated on postoperative day 1; PEDF-5: CSO-SA/PEDF group when treatment was initiated on postoperative day 5).

### Distribution of CSO-SA/PEDF in Model Mice

A polymeric micelle gene delivery system composed of lipid grafted chitosan micelles (CSO-SA) and PEDF (CSO-SA/PEDF) was designed as described previously [31]. Briefly, CSO-SA/PEDF was labeled with fluoresceinisothiocyanate (FITC) by the reaction of the amino group of chitosan and theisothiocyanate group of FITC. After the purity of FITC labeled CSO-SA/PEDF nanoparticles (FITC-CSO-SA/PEDF) was confirmed, 0.1 ml FITC-CSO-SA/PEDF nanoparticles were injected into the model mouse through the vena caudalis. Liver, kidney and endometrial implants were harvested at day 1 (PEDF-1) and day 5 (PEDF-5). Tissue cryosections (5 µm) were cut with a microtome (Leica, Nussloch, Germany) and mounted on slides for examination by fluorescence microscopy (Olympus, AX-70, Japan).

**Figure 6:**
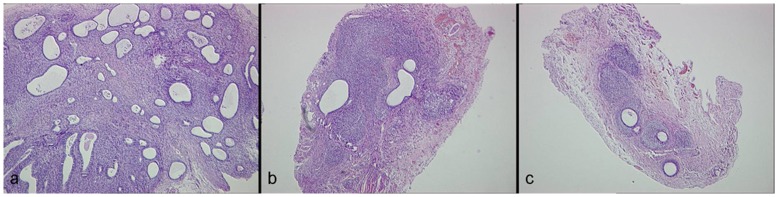
Histopathologic examination of endometriosis lesions (stain: hematoxylin and eosin; magnification × 200). It shows abundant glands and stroma in the control group (a). Gland and stromal structure in PEDF-1(c) and PEDF-5 groups (b) are smaller than that in control group.

**Table 2:** Histopathologic scores, microvessel density and cell apoptosis in endometriotic lesions in the treated and control groups.

Groups	N	HS##	MVD (vWF)	MVD (α-SMA)	Apoptotic cells
Control	4	9.00±0.00	10.35±2.95	8.20±2.93	5.37±2.28
PEDF-1#	6	3.50±1.38*	4.07±1.58*	8.04±2.48	10.37±5.28*
PEDF-5	7	3.85±1.84*	7.10±1.89	8.00±2.39	9.87±4.78*

# PEDF-1 and 5: Mice treated with PEDF gene on day 1 and 5 after transplantation.

## HS = histopathologic score.

* Indicate significant difference (p*<*0.05, versus control).

### Animal Treatment

The mice were divided randomly into three different groups, consisting of nine rats in each group. The first group was given 0.1 ml ( = 5 mg/kg body weight of PEDF) CSO-SA/PEDF via IV injection on postoperative day 1 to investigate the effects of CSO-SA/PEDF on the formation of the implants (PEDF-1). The second group was treated with 0.1 ml ( = 5 mg/kg body weight of PEDF) CSO-SA/PEDF on postoperative day 5 to determine the effects of CSO-SA/PEDF on the development and progression of implanted endometrium. The third group, acted as negative controls, and was injected with 1.5 ml of sterile normal saline through their vena caudalis.

The weight of each mouse and the size of each implant were measured every three days. After two weeks, the implants were measured, photographed and excised. The volume of each lesion was calculated according to the formula: V (volume, mm^3^)  =  (length×width^2^) ×0.5. The endometrium, uterine horn and ovary were dissected and fixed in 10% buffered formaldehyde for hematoxylin and eosin staining and immunohistochemical analysis.

Paraffin-embedded endometrium in control and drug-treated groups was cut into 4 µm sections and observed under light microscopy after H&E staining (Leica, Nussloch, Germany). Histopathologic scores of the implants (HS) were evaluated as described previously [34]. The persistence of epithelial cells in the explants was scored (P) as follows: 3 = well-preserved epithelial layer; 2 =  moderately preserved epithelium with leukocyte infiltrating; 1 =  poorly preserved epithelium (occasional epithelial cells only); and 0 = no epithelium. The intensity of glands (I) ranging from 0 (no glands) to 3 (abundant glands). The histologic scores of the implants were calculated with the formula: HS = PxI.

**Figure 7:**
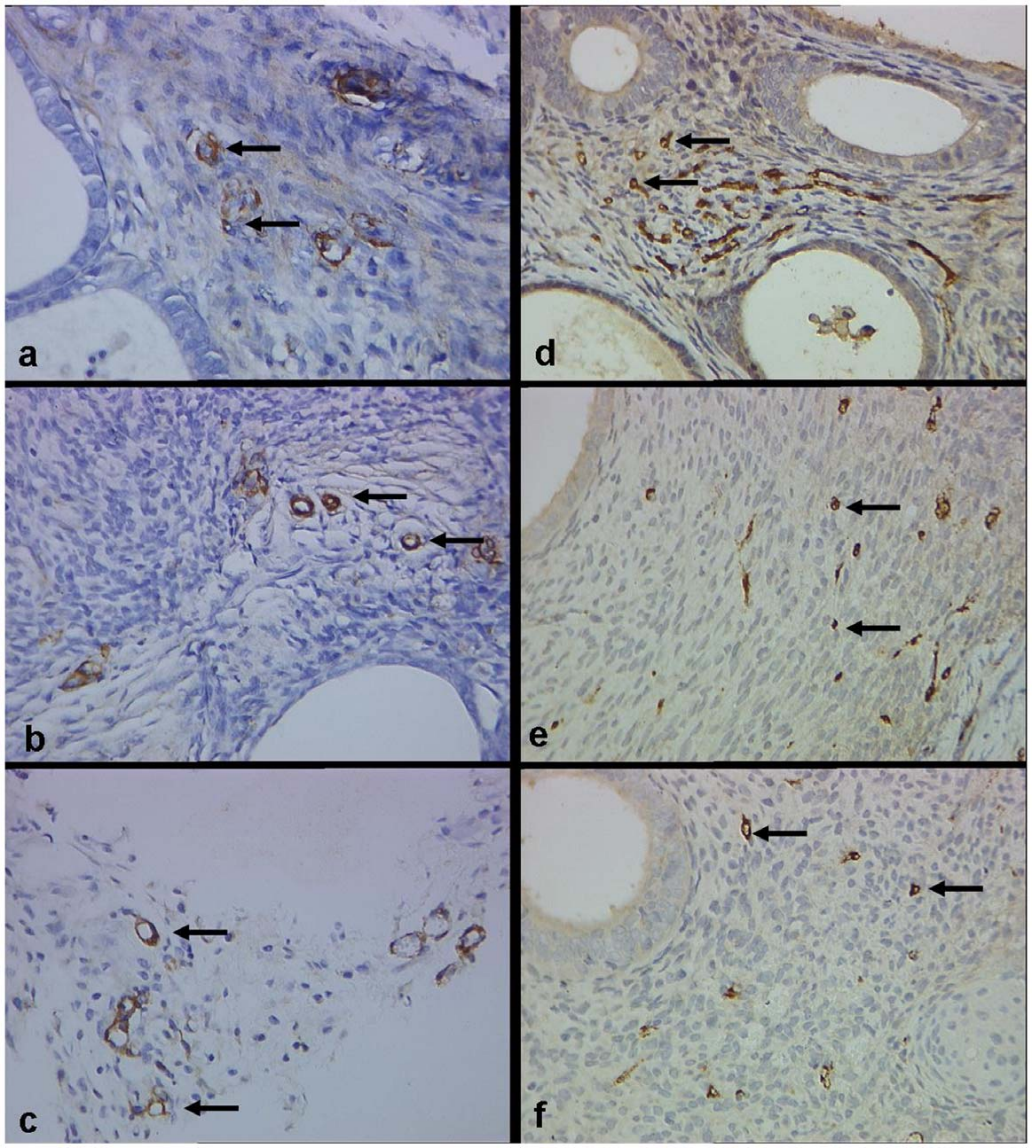
Microvessel density (MVD) of implants stained with α-SMA (a–c) and vWF (d–f) in control, PEDF-5 and PEDF-1 groups (Original magnification ×400). There is no difference between control (a) and PEDF-treated groups (b, c) in α-SMA labeled MVD, while MVD labeled by vWF in the PEDF-1 group (f) is significantly reduced compared to control group (d).

### Immunohistochemical Analysis

To examine the vascular response of CSO-SA/PEDF on endometrium, uterus and ovary, PEDF and VEGF expressions, and microvessel density (MVD) were evaluated. The MVD using Von willebrand factor (vWF) staining described neo-angiogenesis, while MVD labeled by α-smooth muscle actin (α-SMA) described the formation of mature vessels. Primary antibodies included monoclonal mouse VEGF antibody (dilution1∶50; SC-7269; Santa Cruz), monoclonal mouse PEDF antibody (dilution1∶200; M0616; Millpore), monoclonal mouse α-SMA antibody (dilution 1∶2000; M0851; Dako) and polyclonal rabbit vWF antibody (dilution1∶100; M0082; Dako).

Formalin-fixed, paraffin-embedded tissues were cut at 4 µm sections. These sections were deparaffinized and rehydrated in a graded series of ethanol following standard protocols, and then treated with 3% H_2_O_2_ for 10 min to eliminate endogenous peroxidase activity. Sections were immunostained using the primary antibody for 60 min at room temperature. They were then washed in phosphate-buffered saline (PBS) and incubated with Envision-labeled polymer-alkaline phosphates mouse or rabbit for 60 min. The antigen–antibody reaction was visualized using diaminobenzidine as chromogen (DAB, GK346810; Novocastra, UK). After washing, the sections were counterstained with hematoxylin, then dehydrated and mounted.

**Figure 8:**
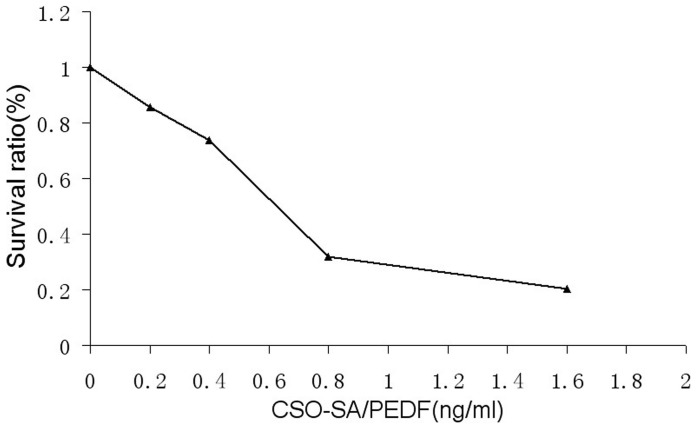
The survival ratio of ECSCs after treatment with CSO-SA/PEDF complex, the number of surviving cells decreased significantly with increasing amounts of CSO-SA/PEDF.

**Figure 9:**
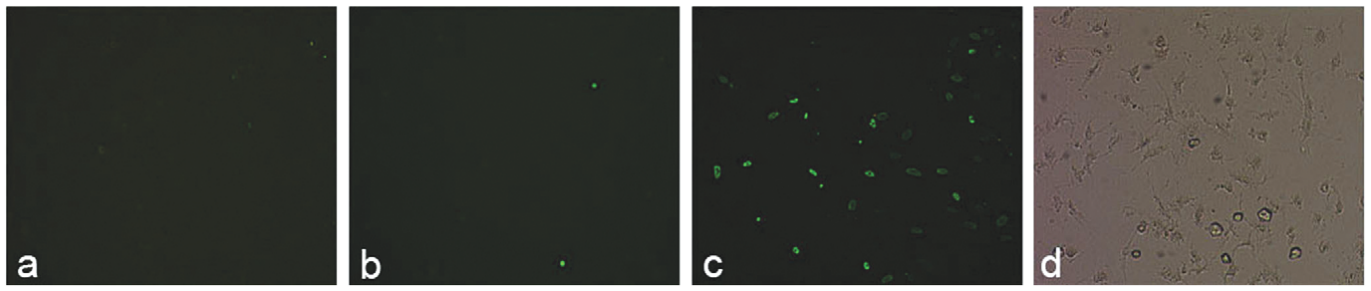
Incubation with CSO-SA/PEDF (24 µl/ml) induced fluorescent TUNEL-positive changes in ECSC nuclei after 48 h and 72 h of treatment. It shows that apoptotic cells in ECSC (d) increases significantly after 48 h (b) and 72 h (c) of treatment comapred to controls (a). (original magnification ×200).

### Evaluation of Immunohistochemical Staining

A semiquantitative evaluation of immunohistochemical staining for VEGF and PEDF expressions was performed under a light microscope according to the method described by Krajewska et al. [35]. The percentage of positive cells was evaluated: 0 points, 0%; 1point, 1% to 25%; 1point, 26% to 50%; 3 points, 51% to 75%; 4 points, >75%. The staining intensity was rated as: 0 points, no staining; 1 point, weak intensity (equivalent to normal epithelium); 2 points, moderate intensity; 3 points, strong intensity. The product of points for expression and percentage of positive cells was the histologic score.

### Quantification of Microvessel Density (MVD)

The MVD was determined by the number of vWF-positive and α-SMA-positive vessels detected by cytoplasmic brown staining. Any distinct single brown-stained cell or cluster of endothelial cells with or without lumen formation or red blood cells was considered to be a microvessel. The sections were scanned under low-power light microscopy (×100) magnification to identify “hot spots” (area stained most intensely). The number of labeled microvessels was calculated over 5 fields at high power (×400). The average vessel count of five different fields was taken as the MVD of the sample.

### Human Endometriotic Stromal Cells Culture

Ovarian endometriotic tissues were obtained from 21 patients undergoing ovarian cystectomy for ovarian endometriosis cysts (mean age 30.05, range 25–37 years, mean parity 0.81, range 0–2 children). All patients had a history of regular menstrual cycles and none received hormonal therapy for six months before surgery.

Isolation of endometriotic stromal cells (ESCs) was performed as previously described [36], [37]. Briefly, endometrium was mechanically dispersed with a scalpel, and then enzymatically digested with 0.1% collagenase type 1 and 0.05% DNAse. Centrifugal setting and filtration were used to separate out the ESCs. The cells were cultured in 6-well plates with Dulbecco’s modified Eagle Medium/F-12 (DMEM/F12) (1∶1) containing 10% fetal calf sera. Immunohistochemical analysis of vimentin (V9, 1∶200, Dako) expression was performed to confirm the purification of the isolated endometriotic stromal cells.

### Intervention of Endometriotic Stromal Cells

The ESCs grown to confluence were detached with trypsin and incubated in 24-well plates at a density of 10^6^ cells per well. Twenty-four hours later, ESCs were treated with 0.1 ml FITC-CSO-SA/PEDF nanoparticles containing 100 ug PEDF, and harvested after 1 h, 4 h, 8 h and 16 h incubation respectively for fluorescence microscope observation (Olympus, AX-70, Japan).

### Cell Proliferation and Viability

Cell proliferation and viability of ESCs were evaluated by a modified 3-[4, 5-dimethylthiazol-2-yl]-2, 5-diphenyltetrazolium bromide (MTT) assay. Briefly, 10^5^ cells were plated in 96-well plates at 37°C and incubated for 24 hours. The ESCs were incubated with 100 µl MTT (5 mg/ml) for 4 h after treatment with different concentrations of CSO-SA/PEDF (0, 0.2 ng/ml, 0.4 ng/ml, 0.8 ng/ml and 1.6 ng/ml) for 48 hours. After 4 hours, the medium was aspirated and 150 µl of DMSO dissolved the MTT formazan crystals. Absorbance of formazan product was measured under a universal microplate reader at wavelength 490 nm, and the ratio of treated cells/untreated cells was calculated as the survival ratio.

### Cell Apoptosis

To evaluate the effect of PEDF on ESCs, apoptosis was quantified by terminal deoxynucleotidyl transferase (TdT)-mediated dUTP nick-end labeling (TUNEL) assay (NO11684 817910, Roche, Indianapolis, IN) according to the manufacturer’s instructions. Detection of apoptosis in ESCs was carried out after CSO-SA/PEDF treatment (16 µl, 2 mg/ml) for 48 h and 72 h. The apoptosis of ESCs was observed under fluorescence microscope at × 200 magnifications (Olympus, AX-70, Japan). Each study was repeated four times.

### Immunocytochemistry Analysis

The ESCs were treated with 16 µl CSO-SA/PEDF (2 mg/ml) for 12 hours, and then medium containing CSO-SA/PEDF complex was aspirated before further 36 hours incubation. The slides were fixed with 4% paraformaldehyde-PBS and primary antibody PEDF (MAB1059, Chemicon, 1∶150 dilutions) and VEGF (SC-7269, Santacruz, 1∶50 dilution) were used for immunocytochemistry analysis according to the manufacturer’s instructions. Positive cells showed reddish brown color while weak positive cells showed light brown color. Staining intensity was graded using a 4-stage scale: negative (−), weakly positive (+), moderately positive (++), and strongly positive (+++). All experiments were repeated four times.

### Statistical Analysis

Data were expressed as Mean ± standard error, and statistical analysis was performed by Student’s t-test and One-Way analysis of variance (ANOVA). P values <0.05 were considered to be statistically significant in all cases.

## Results

### Distribution of PEDF Gene *in vivo* and *in vitro*

After the FITC labeled CSOSA/PEDF were injected to the model mice by vena caudalis for 1, 4, 24 and 48 h, the strong green fluorescence was observed in test tissues at first 1 h, and the fluorescence intensity was brightest in endometriotic lesions. The weak fluorescence was also observed in the uterus and ovaries, but almost no fluorescence was seen in the internal organs (Fig. 1). In ESCs, the green fluorescence appeared in the cytoplasm and nucleus, and its intensity was increased in a time-dependent way (Fig. 2).

### Expression of PEDF Protein *in vivo* and *in vitro*

Immunohistochemical staining showed that PEDF was mostly immunostained in the cytoplasm of glandular epithelial cells, and in stromal cells of the lesions (Fig. 3). When model mice were treated with PEDF gene on day 1 or 5 after transplantation, PEDF protein expression in endometriotic lesions was both significantly increased than it was in the control group ((P<0.05, Table 1 and Fig. 2), indicating that PEDF gene can express protein in endometriotic lesions. However, PEDF protein expression in the uterus and ovaries showed no differences between the treated and control groups (P>0.05), suggesting that PEDF gene may do no harm to the internal reproductive organs. In vitro, there was no expression (-) of PEDF protein in ESCs by using immunocytochemical staining in control group, while a strong positive expression (+++) of PEDF protein was observed in ESCs when treated with PEDF for 48 h (Fig. 4), indicating that PEDF protein expression was up-regulated when PEDF gene treatment.

### Inhibitory Effect of PEDF on Human Endometrial Implants in Nude Mice

Two weeks after transplantation, human endometrial implants developed into cystic lesions, and some formed typical ovoid, fluid-filled foci in mice. Success rate of the endometrial implants on day 1 and 5 after transplantation treated with PEDF (PEDF-1 & PEDF-5) was 77.8% and 100%, respectively, while it was 100% in the control group. The mean volume of the implants was 4.92±3.09 mm^3^ and 9.93±5.81 mm^3^ in the PEDF-1 and PEDF-5 groups, and 29.86±10.05 mm^3^ in the control group (Table 1, Fig. 5). The volume of the implants was both significantly lower in the PEDF-1 and PEDF-5 groups than that in the control group (P<0.05), but no significant difference between the PEDF-1 and the PEDF-5 groups was found (P>0.05, Table 1).

Histologically, the implanted tissues showed typical features of endometriosis including endometrial-type glands and stromal structure. In the control group the lesions possessed abundant epithelial glands and stromal structures. After PEDF treatment, glands and stroma atrophied and degenerated with some tissues exhibiting structural damage to the glands, stromal fibrosis and necrosis (Fig. 6). Histopathologic scores in the implants were 3.50±1.38 and 3.85±1.84 in the PEDF-1 and PEDF-5 groups, which were both significantly lower than that in the control group (9.00±0.00, P<0.05), but no difference between the PEDF-1 and PEDF-5 groups was found (P>0.05, Table 2). In addition, the apoptotic index was both significantly higher in the PEDF-1 and PEDF-5 groups (10.37±5.28 and 9.87±4.78, respectively) than that in the control group (5.37±2.28, P<0.05), but no difference between the PEDF-1 and PEDF-5 groups (P>0.05, Table 2).

VEGF was also immunostained in the cytoplasm of glandular epithelial cells and in stromal cells in endometriotic lesions (Fig. 3), vWF-positive and α-SMA-positive vessels were distributed throughout the stroma of endometriotic lesions and concentrated around the glands (Fig. 7). The MVD labeled by vWF and VEGF expression both significantly decreased when treated with PEDF. Scores of VEGF expression and MVD labeled by vWF were both significantly lower in the PEDF-1 group than that of in the control group (P<0.05), although no difference between the PEDF-5 and the control groups was found (P>0.05; Table 1, 2; Fig. 3, 7). In addition, no significant differences of MVD by using α-SMA staining between the treated groups and the control group were found (P>0.05, Table 2 and Fig. 7).

### Inhibitory Effect of PEDF on ESCs *in vitro*

The growth of ESCs was markedly inhibited in dose-dependent way when treated PEDF. The survival ratio of ESCs was 100%, 85.7%, 73.7%, 31.8% and 20.3% when treated by CSO-SA/PEDF with a dose of 0 ng/ml, 0.2 ng/ml, 0.4 ng/ml, 0.8 ng/ml and 1.6 ng/ml, respectively (Fig. 8). The IC_50_ value of CSO-SA/PEDF against ESCs was 0.864 ng/ml. Moreover, the survival ratio of ESCs was decreased in a time-dependent way when treated with 1.0 ng/ml CSO-SA/PEDF. The percentage of apoptotic cells was 5.7% when treated with CSO-SA/PEDF for 48 h, but the number of apoptotic cells was significantly increased after incubation with CSO-SA/PEDF for 72 h (P<0.05), and the percentage of apoptotic cells was 64.9% (Fig. 9). Similar results were obtained in the repeated experiments. In addition, VEGF protein expression (++) in ESCs was strongly immunostained before PEDF treatment, but only a weak VEGF-positive expression (+) in ESCs was observed after PEDF treatment (Fig. 4), indicating that VEGF expression in ESCs is down-regulated when treated with PEDF gene.

## Discussion

The key to targeted gene therapy for endometriosis is to determine if the targeted gene can reach the site of endometriotic lesions, and produce a functional protein to exert its effects within the lesions. In this study, we showed that PEDF gene mediated by CSO-SA could penetrate the lesions and ovarian endometriotic stromal cells. PEDF protein expression was up-regulated in vivo and in vitro when treated with PEDF gene, suggesting that the transported PEDF gene can be translated into PEDF protein. Although PEDF gene could be distributed to the uterus and ovaries, yet, no changes in protein expression were observed between the PEDF treated and control groups. This indicates a fact that PEDF protein may only be expressed in the targeted tissues, and thus implying that PEDF gene therapy would do no harm to the internal reproductive organs.

Angiogenesis is believed to be a prerequisite for the formation and development of endometriosis [4], [5]. As a natural inhibitor of angiogenesis, PEDF may be more active against endometriotic lesions at the formation stage [26], [30]. Actually, many anti-angiogenic factors including COX-2 inhibitors used to treat endometriosis in animal models exert their effects at this stage [6], [7]. In this study, we used PEDF gene to treat endometriosis at the formation and development stages. We chose PEDF gene treatment on day 1 and 5 after transplantation, which is arbitrarily thought to be at the formation and development stages. In fact, our preliminary experiments and previous studies by others have confirmed that the implants had formed cysts and exhibited a typical endometrial-like glands and stromal structure at 5 days after implantation [38].

Our results showed that PEDF gene treatment on day 1 and 5 after transplantation significantly decreased the size of the implants in nude mice, and caused atrophy and regression of the implanted human endometrium [31]. However, the above-mentioned changes showed more obvious on day 1 compared to those on day 5. The reason may be that there are more new blood vessels in endometriotic lesions at the formation stage (day 1), and thus facilitating PEDF action. Another reason is that the treatment period was shorter on day 5 (9 days) than it was on day 1 (13 days). Our in vitro results showed that the growth of endometrotic stromal cells was inhibited by PEDF treatment in a time-dependent way as well as in a dose dependent way. These observations suggest that PEDF gene therapy may be used as an effective treatment approach for endometriosis, although the time and dose of PEDF gene for the treatment of endometriosis need to be further investigated.

The mechanisms by which PEDF inhibits the growth of endometriotic cells are still uncertain. Our in vivo results showed that PEDF treatment caused a reduction in MVD labeled by vWF but not by α-SMA, and decrease in VEGF expression in the implants in nude mice, suggesting that PEDF gene is active against neo-angiogenesis rather than pre-existing mature blood vessels in endometriotic lesions [21]. Our results also showed that the apoptotic index of ESCs was increased in vitro and vivo when treated with PEDF, and that PEDF protein expression was strongly immunostained in the cytoplasm and nucleus of ESCs (Fig. 4). This indicates that PEDF inhibits the growth of endometriotic cells not only by anti-neovascularization but also through the apoptosis of endometriotic cells themselves.

It has been suggested that VEGF can induce angiogenesis, and play an important role in the formation and development of endometriosis [39], [40], and thus VEGF inhibitor can be used as a treatment for endometriosis [41]. However, PEDF is more potent than any other endogenous inhibitor of angiogenesis, and its inhibitory activity is twice more than that of angiostatin, and seven times more than that of endostatin [17]–[20]. Moreover, PEDF suppresses endothelial cell migration induced by other angiogenic factors such as VEGF [18]–[20]. Obviously, PEDF, a natural inhibitor of angiogenesis, is a very suitable for the treatment of angiogenic diseases. In this study we found that PEDF protein expression in the implants was significantly increased both on day 1 and 5, while VEGF protein expression was only significantly decreased both on day 1. This further supports a fact that PEDF inhibits the growth of endometriotic cells not only by anti-neovascularization but also through the apoptosis of endometriotic cells themselves. Nevertheless, VEGF expression was decreased in vitro and vivo when PEDF treatment, suggesting an important role of VEGF playing in the pathogenesis of endometriosis [29], [30], [39], [40]. Apparently, the adjustment of an imbalance of VEGF and PEDF may be an important mechanism of PEDF gene therapy for endometriosis. However, the mechanisms of how PEDF gene causes decrease in VEGF expression and increase in the apoptosis of endometriotic cells are still needed to be further investigated.

In summary, our results showed that PEDF inhibited the growth of human endometriotic cells in vivo when transplanted to a mouse model, and and in vitro by a process of anti-angiogenesis and apoptosis. However, in this study we only used immunochemical staining to determine the levels of PEDF and VEGF protein. Therefore, further studies including intracyst injection PEDF gene therapy are needed.
